# Use of alcoholic beverages in VA medical centers

**DOI:** 10.1186/1747-597X-1-30

**Published:** 2006-10-19

**Authors:** S Pirzada Sattar, S Faiz Qadri, Mustafa K Warsi, Cordelia Okoye, Amad U Din, Prasad R Padala, Subhash C Bhatia

**Affiliations:** 1 Substance Use Disorders Program, Omaha VA Medical Center, 4101 Woolworth Ave, # 116A, Omaha, Nebraska, USA 68105; 2 Department of Psychiatry, Creighton University School of Medicine, 3528 Dodge Street, Omaha, Nebraska, USA 68131; 3 Department of Psychiatry, Sanford School of Medicine, University of South Dakota, 4400 West 69th Street, Suite 1500, Sioux Falls, South Dakota, USA 57108; 4 Avera Research Institute, 2020 Norton Street S, Sioux Falls, South Dakota, USA 57105; 5 Department of Psychiatry, University of Kansas Medical Center, 3901 Rainbow Blvd., 1012 Olathe, Kansas City, Kansas, USA 66160; 6 Department of Psychiatry, University of Nebraska College of Medicine, 988470 Nebraska Medical Center, Omaha, Nebraska, USA 68198-8470

## Abstract

**Background:**

Benzodiazepines are the first-line choice for the treatment of alcohol withdrawal syndrome. However, several hospitals continue to provide alcoholic beverages through their formulary for the treatment of alcohol withdrawal. While there are data on the prevalence of this practice in academic medical centers, there are no data on the availability of alcoholic beverages at the formularies of the hospitals operated by the department of Veteran's Affairs.

**Methods:**

In this study, we surveyed the Pharmacy managers at 112 Veterans' Affairs Medical Centers (VAMCs) to ascertain the availability of alcohol on the VAMC formularies, and presence or lack of a policy on the use of alcoholic beverages in their VA Medical Center.

**Results:**

Of the pharmacy directors contacted, 81 responded. 8 did not allow their use, while 20 allowed their use. There was a lack of a consistent policy across the VA medical centers on availability and use of alcoholic beverages for the treatment of alcohol withdrawal syndrome.

**Conclusion:**

There is lack of uniform policy on the availability of alcoholic beverages across the VAMCs, which may create potential problems with difference in the standards of care.

## Background

Alcohol Use Disorders are a major public health problem in the US. Each year over a million patients are admitted to hospitals across US, requiring medical treatment for preventing and treating alcohol withdrawal [1,2]. In the VA Medical system, more than half of the admissions to the inpatient psychiatric units are related to alcohol and drug use [3].

While benzodiazepines are thought be the most appropriate treatment for prevention/treatment of alcohol withdrawal [1-5], some advocate the use of alcoholic beverages to prevent seizures [6]. However, others advise against the use of alcoholic beverages because of a low therapeutic window, short half-life, pharmacologic interactions, potential for teratogenicity, and severe medical complications [5,7,8]. Current evidence based practices do not support their use [9], but more than 70% of the 116 US teaching hospitals continue to have alcoholic beverages on their formularies [10,11].

The Department of Veterans Affairs (VA) does not have a central policy regulating the use of alcoholic beverages and individual hospitals have local control over their use. However, there are no data on how many VA hospitals make alcoholic beverages available through their formularies for the treatment of alcohol withdrawal. The objective of this study is to collect data on this practice.

## Results

A total of 112 hospitals were contacted, of which pharmacy directors from 81 (72%) hospitals responded. 10% (n = 8) reported an existing policy against their use, while 25% reported having policy allowing their use (20% {n = 16} with formulary availability, while 5% {n = 4} allowed it but through the non-formulary process).

Approximately, 65% of the VAMCs did not have a policy, 11% (n = 9) made it available on their formulary, 11% (n = 9) made it available through a non-formulary process, while 43% (n = 35) did not provide alcoholic beverages.

Of the 38 VAMCs that made alcoholic beverages available to their physicians, beer was the most widely used/requested beverage (n = 16, 41%) followed by whiskey (n = 6, 15%), and wine (n = 6, 15%). Pharmacy was primarily responsible for obtaining the alcoholic beverages while nursing was responsible for their administration. When asked about their perception of the subspecialty most likely to use alcoholic beverages, the pharmacy directors identified Internists as their most frequent prescribers, followed by Surgeons, Psychiatrists and others. A third of the pharmacy directors suggested that these beverages were used for patient courtesy, while another third thought it was for preventing/treating alcohol withdrawal, while others thought it was part of the meal, or for sedation.

## Discussion

An important finding of this study was the lack of a uniform policy across the VA medical system on the issue of using alcoholic beverages. In the absence of a central VA policy governing their use, individual hospitals have adopted their own policies on the availability of alcoholic beverages which potentially creates a disparity in the care of veterans and may appear to some as a restriction of treatment options.

The use of alcoholic beverages for the treatment of alcohol withdrawal is controversial and debatable from a scientific point of view. It also raises some ethical concerns. Using alcoholic beverages for the treatment of individuals with alcohol dependence might send a conflicting message that their use may be appropriate and not problematic. Further, this may also put the prescribing physicians in the awkward position of using substances that they or their colleagues advocate their patients quit or limit.

While the benefit of using intravenous alcohol for the treatment of some acute conditions is established [6], the empiric data on the benefits of using alcoholic beverages for treating alcohol withdrawal is not so clear. This study had several limitations, including a lack of descriptive data that might provide more information on trends within the VA system on the use of alcoholic beverages. More research is needed on the use of alcoholic beverages so that current medical practices can be evaluated and on the basis of empirical evidence.

## Conclusion

There is no uniform standard on the availability and use of alcoholic beverages at the VA medical centers across the country. This potentially leads to discrepancies in the standard of care for patients in different geographic locations. The authors would suggest that the VA make efforts to have consistent policy on this issue across their medical centers to ensure uniformity in the standard of care offered to veterans.

## Method

The pharmacy directors at all the VA hospitals across the US were contacted and invited to participate in this study. The country is divided into 23 areas within the Veterans Integrated Service Network (VISN). Pharmacy directors at all hospitals within the 23 VISNs were contacted by email first, along with a follow up telephone call for non-responders. Each pharmacy director was invited to participate in this survey and complete a special questionnaire, that was developed by the authors based on the work of Smoger et. al. [9,10]. This questionnaire not only inquired about the use of alcoholic beverages but also collected data about the existence of a policy for its use and further clarified the preferred beverage, and the method of procuring and administering these beverages. We also asked pharmacy directors about heir opinion on which sub-specialties were using alcoholic beverages. The Omaha VA Medical Center's Committee on Human Subjects approved this study. Pharmacy directors were contacted by email, fax and telephone, and invited to participate. Their responses were entered on a database using SPSS and results were computed for descriptive analysis.

## Competing interests

Authors are associated with the Omaha VA medical Center and have worked with local committees to abolish the practice of using alcoholic beverages for the treatment of alcohol withdrawal at their respective institutions.

## Funding

No external sources of funding were utilized for this study.
